# Genetic and immunologic features associated with thrombocytopenia progression and poor prognosis in patients with myelofibrosis

**DOI:** 10.3389/fmed.2024.1461421

**Published:** 2024-11-07

**Authors:** Tong Yoon Kim, Ki-Seong Eom, Ji Yoon Lee, Jong-Mi Lee, Myungshin Kim, Sung-Eun Lee

**Affiliations:** ^1^Department of Hematology, Catholic Hematology Hospital, Yeouido St. Mary’s Hospital, College of Medicine, The Catholic University of Korea, Seoul, Republic of Korea; ^2^Department of Hematology, Catholic Hematology Hospital, Seoul St. Mary’s Hospital, College of Medicine, The Catholic University of Korea, Seoul, Republic of Korea; ^3^Severance Biomedical Science Institute, Yonsei University College of Medicine, Seoul, Republic of Korea; ^4^Department of Laboratory Medicine, Seoul St. Mary’s Hospital, College of Medicine, The Catholic University of Korea, Seoul, Republic of Korea

**Keywords:** myelofibrosis, thrombocytopenia progression, prognosis, ASXL1 mutation, CD45RA + CD4 + T cells

## Abstract

**Introduction:**

Myelofibrosis, which includes primary myelofibrosis (PMF) and secondary myelofibrosis (SMF), can exhibit cytopenic features associated with poor outcomes; however, the underlying mechanisms are unclear. Moreover, characterized by its aggressive nature and limited therapeutic options, myelofibrosis poses a major clinical challenge in hematology. Therefore, in this study, we aimed to identify genetic and immunologic features associated with thrombocytopenia progression and poor prognosis.

**Methods:**

The study involved 226 patients with PMF or SMF, who were categorized into three groups: platelet count ≥ 100 × 10^9^/L (PLT ≥ 100 group; n = 131), progression to thrombocytopenia (PROG group; n = 64), and platelet count < 100 × 10^9^/L (PLT < 100 group; n = 31).

**Results:**

Survival analysis revealed 4-year overall survival rate of 57.7%, 89.4%, and 93.9% for the PLT < 100, PROG, and PLT ≥ 100 groups, respectively. Time-dependent covariate analysis of the PLT ≥ 100 and PROG groups revealed inferior overall survival rate of the PROG group. Multivariate analysis indicated that progression to thrombocytopenia and *ASXL1* and *IDH1* mutations were associated with poor overall survival. Flow cytometry revealed fewer CD45RA^+^CD4^+^ T cells in the PROG group than in the PLT ≥ 100 group. *ASXL1* mutations were more prevalent in the PROG group than in the other groups, correlating with a reduced number of CD45RA^+^CD4^+^ T cells.

**Discussion:**

*ASXL1* mutation and low CD45RA^+^CD4^+^ T-cell counts correlated with progression to thrombocytopenia. Our findings underscore the clinical significance of thrombocytopenia dynamics in MF progression and prognosis, with implications for patient management and therapeutic interventions.

## 1 Introduction

Myelofibrosis (MF) is the most aggressive form of BCR::ABL1-negative myeloproliferative neoplasms (MPNs) and is a clonal hematological disorder characterized by the overproduction of differentiated hematopoietic cells. The incidence of MF ranges from 0.1 to 1 per 100,000 individuals per year, with a median survival of 63 months (1, 2). According to the 2016 World Health Organization (WHO) classification, MF can be classified as primary myelofibrosis (PMF) and secondary myelofibrosis (SMF), PMF is further divided into overt primary myelofibrosis or prefibrotic myelofibrosis (pre-PMF). PMF develops independently, whereas SMF develops secondary to other pre-existing disorders, such as polycythemia vera (PV) and essential thrombocythemia (ET).

Myelofibrosis can become life-threatening owing to various pathological conditions, such as thrombosis, infection, and leukemic transformation (3). The progression of MF is typically associated with the development of cytopenic features, such as thrombocytopenia, and the prevalence of thrombocytopenia at the time of MF diagnosis ranges from 11% to 26% (4, 5). MF with cytopenic features is associated with poor outcomes and difficulties adhering to ruxolitinib; however, the available information is limited to diagnostic data (6, 7). Cytopenic progression is a lifelong process, and hence, the prognostic value of cytopenic progression should be evaluated using a time-dependent model (6–9). Therefore, in this study, we aimed to evaluate the prognostic value of thrombocytopenia as a time-dependent covariate in patients with pre-PMF, overt PMF, and SMF and to identify genetic and immunologic features associated with the progression to thrombocytopenia and poor prognosis.

## 2 Materials and methods

### 2.1 Study patients and their classification criteria

This retrospective study involved 226 patients aged ≥ 18 years who were diagnosed and treated for MF at Seoul St. Mary’s between December 2001 and August 2021. Myelofibrosis diagnosis was confirmed and classified based on the 2016 WHO classification (10). Secondary MF was defined as PV or ET progressed to post-PV or post-ET (11).

This study was approved by the relevant institutional review board (KC22RISI0120) and was conducted in accordance with the tenets of the Declaration of Helsinki. The requirement of obtaining patient consent was waived owing to the retrospective nature of the study.

### 2.2 Molecular and cytogenetic analyses

DNA samples were obtained from bone marrow aspirate samples, irrespective of progressive MPN disease. Conventional bone marrow karyotyping was performed using routine techniques, and karyotypes were interpreted according to the International System for Human Cytogenomic Nomenclature (ISCN) 2016 guidelines (12). Next-generation sequencing was performed using a customized myeloid panel (SM panel) (13). The SM panel comprised 87 genes that frequently exhibit mutations in patients with MPN. Target capture sequencing was analyzed using a customized target kit (3039061; Agilent Technologies). DNA libraries were prepared according to the manufacturer’s instructions and sequencing was conducted using an Illumina HiSeq4000 platform. Mutations were defined as variants with > 5% variant allele frequencies (VAFs). Janus Kinase 2 *(JAK2)*, calreticulin (*CALR)*, and MPL proto-oncogene (*MPL)* mutations were considered positive at a VAF below 5% owing to a low allele burden. All mutations were manually identified using the Integrative Genomic Viewer (14).

For multi-parameter flow cytometric analysis, the following antibodies were used: CD56-BUV395 (BD Pharmingen, Franklin Lakes, NJ, USA; catalog # 563554), CD16-BUV395 (BD Pharmingen; catalog # 563785), CD3-FITC (BioLegend, CA, USA; catalog # 317306), CD4-APC (BioLegend; catalog # 300552), CD8α-PerCP (BioLegend; catalog # 344710), HLA-DR-PE (BioLegend; catalog # 307606), CD45RA-PB (BioLegend; catalog # 304123), CD25-APC-Cy7 (BD Pharmingen; catalog # 557753), and CD127-PE-Cy7 (BD Pharmingen; catalog # 560822). Fixable viability stain was procured from BD Pharmingen (catalog # 564996). After fixation with 2% paraformaldehyde, cells were acquired and analyzed using a BD FACSCanto II flow cytometry system (BD Bioscience, Franklin Lakes, NJ, USA).

### 2.3 Classification, scoring systems, and statistical analysis

**FIGURE 1:**
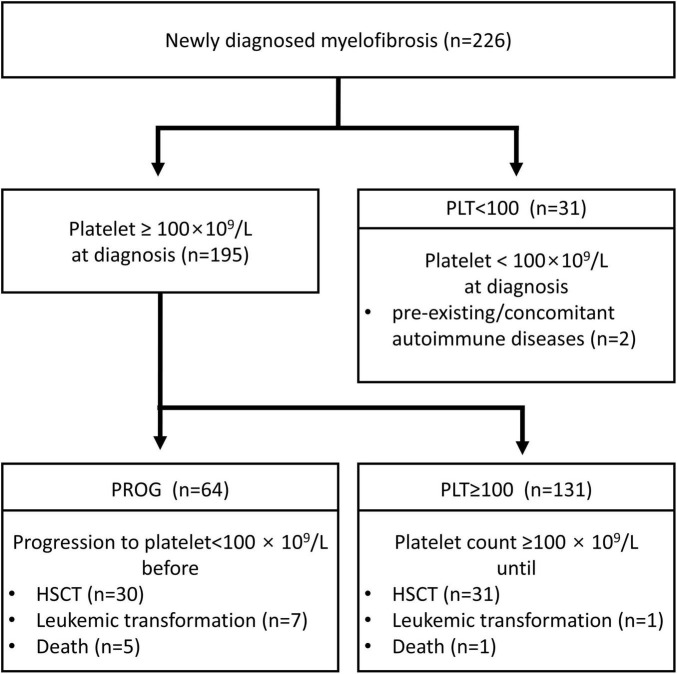
CONSORT flow diagram. PLT ≥ 100, diagnosis at platelet count ≥ 100 × 10^9^/L; PROG, progression to a platelet count of < 100 × 10^9^/L; PLT < 100, diagnosis at platelet count < 100 × 10^9^/L; HSCT, hematopoietic stem cell transplantation.

Patients were classified according to their thrombocytopenia status: PLT ≥ 100 group (n = 131), with a platelet count of 100 × 10^9^/L or more at diagnosis; PROG group (n = 64), showing progression to thrombocytopenia during follow-up; and PLT < 100 group (n = 31), with a platelet count of < 100 × 10^9^/L at diagnosis. Thrombocytopenia caused by pre-existing/concomitant autoimmune diseases was observed in two patients. The CONSORT diagram is presented in Figure 1. The rationale for using a platelet count cut-off of 100 × 10^9^/L was based on the risk factor defined in the Dynamic International Prognostic Scoring System-plus (DIPSS-plus) (15).

To conservatively define the PROG group and ensure that thrombocytopenia was not influenced by treatments such as JAK2 inhibitors, we collected complete blood count profile results and treatment history from diagnosis to the last follow-up. Hematological parameters, symptom scores, and splenomegaly were assessed every 3 months and as needed for early evaluation. For patients receiving JAK2 inhibitors, dose modifications were performed following the approved label. Patients were required to be on a stable daily dose for at least 3 months and have no discernible secondary reasons for thrombocytopenia to exclude thrombocytopenia from secondary causes (such as treatment and infection).

Risk analysis of clinical variables was performed using the International Prognostic Scoring System (3), DIPSS (16), DIPSS-plus (15), Mutation-Enhanced International Prognostic Score System for Transplantation (MIPSS70) (17), Mutation and Karyotype-Enhanced International Prognostic Scoring System for Primary Myelofibrosis (MIPSS70+Ver2) (18), and Myelofibrosis Secondary to PV and ET-Prognostic Model (MYSEC-PM) (19). Clinical and molecular characteristics were compared using the chi-square test or Fisher’s exact test for categorical variables and the two-sample *t*-test or Mann–Whitney *U* test for continuous variables. Results with *p*-value < 0.05 were considered statistically significant.

Overall survival (OS) was defined as the duration from the date of diagnosis or platelet count < 100 × 10^9^/L until death from any cause. In subgroup analysis of OS [except the thrombocytopenia group (PLT ≥ 100 and PROG groups)], proportionality assumption was tested by adding a time-dependent covariate for each factor; the effects of progression to a platelet count of < 100 × 10^9^/L on the preceding OS were assessed using a time-dependent covariate in the final multivariate model. Survival analysis was performed using the Kaplan–Meier method, and groups were compared using the log-rank test. The Cox proportional hazards model was used for univariate and multivariate OS analyses. Variables with a *p*-value of < 0.10 determined using univariate analysis were considered for multivariate analysis. The cumulative incidence of leukemia (CIL) and non-leukemia mortality (NLM) was calculated from the date of diagnosis. For CIL, leukemia was assessed as an uncensored event, and death without progression was considered a competing risk. NLM was defined as death without leukemia and considered a competing risk for leukemic transformation. The CIL and NLM were estimated using the cumulative incidence of competing events.

All statistical analyses were performed using the R software (version 4.0.6; R Foundation for Statistical Computing, Vienna, Austria).

## 3 Results

### 3.1 Clinical, cytogenetic, and molecular features

The clinical and cytogenetic characteristics of the study patients are summarized in Table 1. We divided patients with three groups: platelet count 100 × 10^9^/L or more (PLT ≥ 100 group; n = 131 patients, 58%), progression to thrombocytopenia (PROG group; n = 64, 28.3%), and platelet count < 100 × 10^9^/L (PLT < 100 group; n = 31, 13.7%). The PLT ≥ 100 group had fewer patients with PMF than the other groups. Anemia and the requirement for red blood cell transfusion were more prevalent in the PLT < 100 group than in the other groups. The *ASXL1* mutation was most frequently observed in the PROG group. The incidence of triple-negative MF was higher in the PLT < 100 group than in the other groups.

**TABLE 1:** Baseline clinical and cytogenetic characteristics.

Variable	All (*n* = 226)	PLT ≥ 100 (*n* = 131)	PROG (*n* = 64)	PLT < 100 (*n* = 31)	PLT ≥ 100 vs. PROG	PROG vs. PLT < 100	PLT ≥ 100 vs. PLT < 100	*p*-value
Myelofibrosis, *n* (%)					<0.001	0.131	<0.001	<0.001
SMF	66 (29.2)	46 (35.1)	17 (26.6)	3 (9.7)				
PMF	120 (53.1)	51 (38.9)	44 (68.8)	25 (80.6)				
Pre-PMF	40 (17.7)	34 (26.0)	3 (4.7)	3 (9.7)				
Age > 65 years, *n* (%)	58 (25.7)	31 (23.7)	17 (26.6)	10 (32.3)	0.792	0.738	0.447	0.604
Anemia, n (%)	111 (49.1)	49 (37.4)	33 (51.6)	29 (93.5)	0.084	<0.001	<0.001	<0.001
Leukocytosis > 25 × 10^9^/L, *n* (%)	27 (11.9)	15 (11.5)	11 (17.2)	1 (3.2)	0.378	0.112	0.296	0.139
Peripheral blast ≥ 1%, *n* (%)	94 (41.6)	46 (35.1)	31 (48.4)	17 (54.8)	0.103	0.714	0.069	0.057
Constitutional symptoms, *n* (%)	111 (49.1)	51 (38.9)	40 (62.5)	20 (64.5)	0.003	> 0.999	0.017	0.002
DIPSS *Unfavorable karyotype	5 (2.2)	1 (0.8)	2 (3.1)	2 (6.5)	0.523	0.832	0.17	0.129
Transfusion history, n (%)	49 (21.7)	1 (0.8)	7 (10.9)	19 (61.3)	0.485	< 0.001	<0.001	< 0.001
Myelofibrosis ≥ 2, *n* (%)	183 (81.0)	18 (13.7)	12 (18.8)	27 (87.1)	< 0.001	0.309	0.144	< 0.001
**Mutation, *n* (%)**								
*JAK2*	115 (50.9)	68 (51.9)	39 (60.9)	8 (25.8)	0.3	0.003	0.016	0.005
*CALR* Type 1/like	39 (17.3)	25 (19.1)	12 (18.8)	2 (6.5)	> 0.999	0.202	0.153	0.230
*MPL*	10 (4.4)	4 (3.1)	2 (3.1)	4 (12.9)	> 0.999	0.165	0.069	0.047
*ASXL1*	65 (28.8)	31 (23.7)	28 (43.8)	6 (19.4)	0.007	0.036	0.782	0.007
*SRSF2*	5 (2.2)	2 (1.5)	2 (3.1)	1 (3.2)	0.84	> 0.999	>0.999	0.712
*EZH2*	5 (2.2)	4 (3.1)	1 (1.6)	0 (0.0)	0.892	> 0.999	0.733	0.534
*IDH1*	3 (1.3)	1 (0.8)	1 (1.6)	1 (3.2)	1	> 0.999	0.832	0.549
*IDH2*	4 (1.8)	2 (1.5)	2 (3.1)	0 (0.0)	0.84	0.816	> 0.999	0.528
*U2AF1Q157*	3 (1.3)	0 (0.0)	3 (4.7)	0 (0.0)	0.06	0.549	NA	0.021

DIPSS, Dynamic International Prognostic Scoring System; n, number; PLT < 100, < 100 × 10^9^/L platelet count group; PLT ≥ 100, 100 × 10^9^/L or more platelet count group; PMF, primary myelofibrosis; pre-PMF, prefibrotic myelofibrosis; PROG, progression to thrombocytopenia with platelet count < 100 × 10^9^/L group; SMF, secondary myelofibrosis; NA, not available. *Unfavorable karyotype: Complex karyotype or one or two abnormalities including +8, −7/ 7q-, i(17q), −5/ 5q-, 12p-, inv(3), or 11q23 rearrangement.

### 3.2 Association between survival outcomes and thrombocytopenia dynamics

**FIGURE 2:**
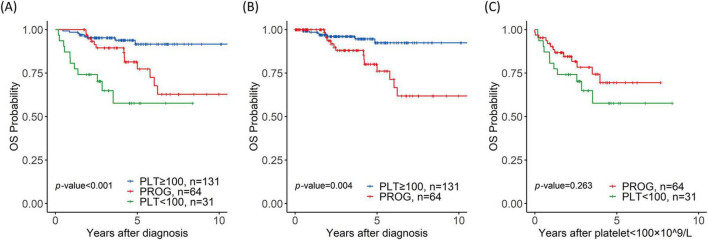
Prognostic value of thrombocytopenia for overall survival (OS). **(A)** Overall survival of the platelet < 100 × 10^9^/L (PLT < 100), progression (PROG), and PLT ≥ 100 × 10^9^/L (PLT ≥ 100) groups; **(B)** OS of the PROG and PLT ≥ 100 groups with time-dependent covariates; **(C)** OS of the PROG and PLT < 100 groups from the time when the platelet count dropped to < 100 × 10^9^/L.

With a median follow-up of 4 years (range, 1.3–21 years), the 4-year OS rates was 57.7%, 89.4%, and 93.9% for the PLT < 100, PROG, and PLT ≥ 100 groups, respectively (PLT < 100 vs. PROG, *p* = 0.014; PROG vs. PLT ≥ 100, *p* = 0.009; PLT < 100 vs. PLT ≥ 100, *p <* 0.001) (Figure 2A). The time points of changes in platelet count threshold and the starting times of JAK2 inhibitor therapy are described in Supplementary Figure 1. In the entire patient cohort, 76 patients underwent allogeneic hematopoietic stem cell transplantation: 15 patients in the PLT < 100 group, 30 in the PROG group, and 31 in the PLT ≥ 100 group.

Time-dependent covariate analysis of the PLT ≥ 100 and PROG groups revealed that progression to thrombocytopenia was associated with a short OS (*p* = 0.004) (Figure 2B). The univariate analysis results are shown in Table 2. The multivariate analysis revealed that progression to thrombocytopenia (*p* = 0.042, hazard ratio (HR) = 7.7, 95% confidence interval (CI) = 1.04–7.70), *ASXL1* mutation (*p* = 0.041, HR = 9.91, 95% CI = 1.05–9.91), and *IDH1* mutation (*p* = 0.02, HR = 75.6, 95% CI = 5.19–1103) were associated with poor OS. Mortality in the PROG group was largely owing to transformation to leukemia, with the PROG group exhibiting a higher CIL (*p* = 0.013, HR = 14, 95% CI = 1.73–113) than the PLT ≥ 100 group (Supplementary Figure 2A).

**TABLE 2:** Overall survival estimation using univariate and multivariate analyses of clinical/genetic variables in patients with MF.

	Univariate		Multivariate	
**Variable**	**95% CI**	***p*-value**	**95% CI**	***p*-value**
PROG vs. PLT ≥ 100	3.73 [1.51–9.25]	0.004	7.70 [1.04–7.70]	0.042
PMF vs. SMF	1.85 [0.74–4.59]	0.188		
Age (years) at diagnosis ≥ 65 vs. < 65	3.49 [1.45–8.39]	0.005	4.35 [0.61–4.35]	0.327
Hemoglobin (g/dL) < 10 vs. ≥ 10	4.00 [1.55–10.3]	0.004	7.62 [0.87–7.62]	0.088
White blood cells (10^9^/L) > 25 vs. ≤ 25	2.42 [0.94–6.24]	0.068	1.57 [0.10–1.57]	0.189
Peripheral blast (%) > 1 vs. ≤ 1	4.56 [1.76–11.8]	0.002	7.70 [0.79–7.70]	0.121
Constitutional symptoms, yes vs. no	1.89 [0.78–4.57]	0.156		
DIPSS *Unfavorable karyotype vs. other karyotypes	4.06 [0.54–30.6]	0.175		
PRC transfusion dependent vs. independent	3.64 [1.45–9.10]	0.006		
Myelofibrosis ≥ 2 vs. < 2	4.91 [0.66–36.7]	0.121		
*CALR* type 1/like mutation vs. wild-type	0.17 [0.02–1.28]	0.086	1.29 [0.02–1.29]	0.085
*ASXL1* mutation vs. wild-type	4.89 [1.97–12.1]	< 0.001	9.91 [1.05–9.91]	0.041
*SRSF2* mutation vs. wild-type	7.58 [1.73–33.2]	0.007	24.4 [0.57–24.4]	0.17
*EZH2* mutation vs. wild-type	0.00 (0.00 to Inf)	0.998		
*IDH1* mutation vs. wild-type	10.1 [1.30–78.8]	0.027	75.6 [5.19–1103]	0.002
*IDH2* mutation vs. wild-type	2.29 [0.31–17.1]	0.419		
*U2AF1*Q157 mutation vs. wild-type	9.75 [2.21–43.1]	0.003	11.90 [0.45–11.90]	0.318

CI, confidence interval; DIPSS, Dynamic International Prognostic Scoring System; PMF, primary myelofibrosis; PLT ≥ 100, 100 × 10^9^/L or more platelet count group; vs., versus; PRC, packed red cells; PROG, progression to thrombocytopenia with platelet count < 100 × 10^9^/L group; SMF, secondary myelofibrosis. *Unfavorable karyotype: Complex karyotype or one or two abnormalities including +8, −7/ 7q-, i(17q), −5/ 5q-, 12p-, inv(3), or 11q23 rearrangement.

A comparison of the PROG and PLT < 100 groups revealed no significant differences in the 4-year OS rate (68.2% vs. 57.7%; *p* = 0.263) (Figure 2C). The median time until progression to thrombocytopenia in the PROG group was 2.1 years; the PLT < 100 group exhibited thrombocytopenia at the time of MF diagnosis. The rate of NLM was higher in the PLT < 100 group than in the PROG group (*p* = 0.008, HR = 3.38, 95% CI = 1.37–8.33) (Supplementary Figure 2B).

### 3.3 Multi-parameter flow cytometric analysis of genomic subgroups in MF

**FIGURE 3:**
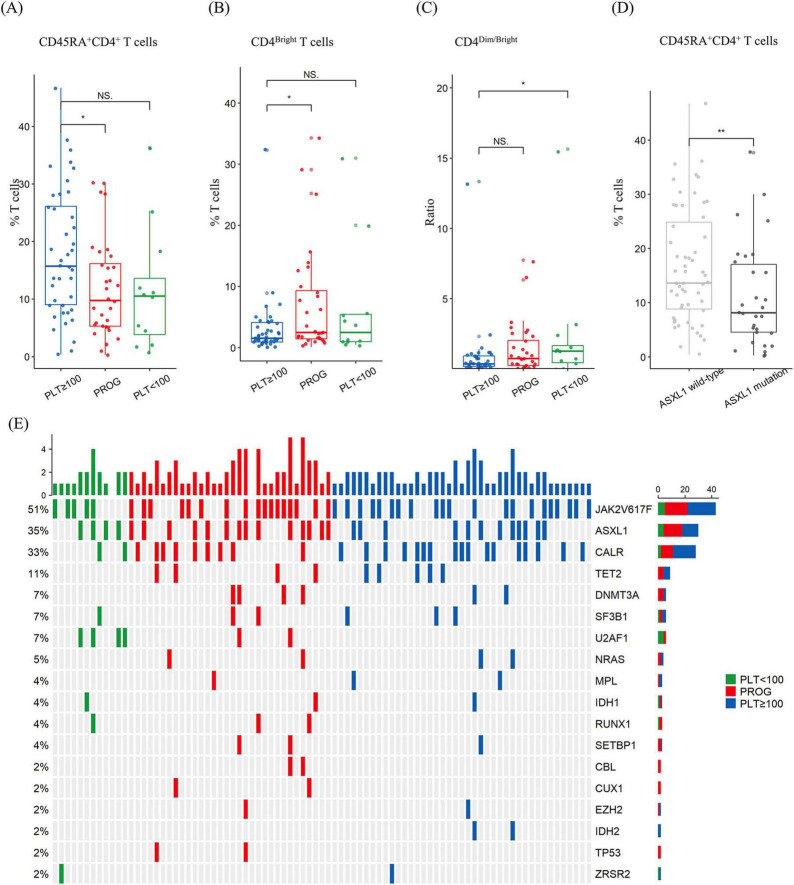
Percentage of T-cell subtypes and mutational spectrum in patients with myelofibrosis according to thrombocytopenia subgroups. **(A)** The progression (PROG) group had a lower proportion of CD45RA^+^CD4^+^ T cells than the platelet ≥ 100 × 10^9^/L (PLT ≥ 100) group. **(B)** The PROG group had a higher proportion of HLA^–^DR^+^CD4^bright^ T cells than the PLT ≥ 100 × 10^9^/L group. **(C)** The platelet < 100 × 10^9^/L (PLT ≥ 100) group had a higher ratio of CD4^dim^ -to-CD4^bright^ T cells than the PLT ≥ 100 group. **(D)** The *ASXL1*-mutation group had a lower proportion of CD45RA^+^CD4^+^ T cells than the wild-type group. **(E)** Heatmap of mutations. NS, *p* > 0.05; *, *p* < 0.05; **, *p* < 0.01.

Disease progression may depend on T-cell activation and expansion. Therefore, we performed multi-parameter flow cytometric analysis to evaluate the composition of various T-cell subsets in the samples collected from 85 patients: 41 samples from the PLT ≥ 100 group, 32 from the PROG group, and 12 from the PLT < 100 group. The prevalence of CD45RA^+^CD4^+^ T cells was lower in the PROG group than in the PLT ≥ 100 group (11.8% ± 8.5% vs. 17.8% ± 11.2%; *p* = 0.014) (Figure 3A). The PROG group exhibited a higher proportion of CD4^bright^ T cells than the PLT ≥ 100 group (7.0% ± 8.7% vs. 3.1% ± 5.1%; *p* = 0.029) (Figure 3B). Furthermore, the PLT < 100 group exhibited a greater CD4^dim^-to-CD4^bright^ ratio than the PLT ≥ 100 group (2.4 ± 4.3 vs. 0.9 ± 2.1; *p* = 0.027) (Figure 3C). No significant differences were observed among the three groups for the other cell types (Supplementary Figure 3 and Supplementary Table 1).

*ASXL1* mutations were detected in 30 of the total samples analyzed using flow cytometry, and the *ASXL1*-mutated group exhibited a lower incidence of CD45RA^+^CD4^+^ T cells than the non-mutated group (16.7% ± 10.6% vs. 11.0% ± 9.6%; *p* = 0.015) (Figure 3D). No significant differences were observed between the *ASXL1*-mutated and non-mutated groups for the other cell types. A detailed comparison of the genomic alteration is shown in Figure 3E.

## 4 Discussion

In this study, we investigated the clinical effect of dynamic thrombocytopenia in patients with MF. Although MPN diseases are commonly identified as clonal proliferative disorders, some patients with MF exhibit a cytopenic phenotype that resembles bone marrow failure. In PMF, thrombocytopenia (defined as platelet count < 100 × 10^9^/L) is an independent predictor of poor survival, according to the DIPSS-plus (15) and MIPSS70/-plus (18) scores. In SMF, thrombocytopenia (defined as platelet count < 150 × 10^9^/L) is associated with a short OS, as reflected by the MYSEC-PM scores (19). In pre-PMF, cytopenia (including leukopenia, anemia, and thrombocytopenia) negatively affects OS (6), and thrombocytopenia allows discrimination between PMF and pre-PMF (20, 21). The association between inferior survival outcomes and thrombocytopenia progression may be attributed to the leukemic transformation in patients with MF (4, 22). In addition, cytopenic MF is associated with a high possibility of treatment failure with ruxolitinib and with worse outcomes (7).

Here, we demonstrated that progression to thrombocytopenia has prognostic value in patients with a platelet count of ≥ 100 × 10^9^/L. Following the onset of thrombocytopenia, all groups exhibited similar survival outcomes. We identified *ASXL1* mutation and low CD45RA^+^CD4^+^ T-cell prevalence as potential aggravators of thrombocytopenia. CD45RA^+^ T cells are associated with naive T cells (23), and patients with autoimmunity exhibit low CD45RA^+^ T-cell counts (24), which is similar to the trend observed in bone marrow failure syndrome related to autoimmunity. Furthermore, we found greater CD4^bright^ T-cell abundance in the PROG group than in the PLT ≥ 100 group. CD4^bright^ helper T cells exhibit increased interferon (IFN)-γ production (25), which is associated with autoimmune MF progression (26) and *ASXL1*-mediated hematopoiesis (27). Additionally, we observed an increase in the ratio of CD4^dim^-to-CD4^bright^ T cells in the PLT < 100 group compared with that in the PLT ≥ 100 group. CD4^dim^ cells are frequently observed in hemophagocytic syndrome (28), which is also associated with thrombocytopenia. Our results implicate the involvement of an inflammatory process prior to thrombocytopenia progression and suggest that CD4^+^ T-cell status is a potential indicator of progression to thrombocytopenia. Therefore, immunomodulatory drugs such as lenalidomide may be candidates for inhibiting the progression of MF. However, caution should be exerted considering the adverse effects of these drugs, including severe cytopenia (29, 30).

Our results indicated a higher incidence of *ASXL1* mutation in the PROG group than in the non-thrombocytopenia group, which corresponds to the findings of a previous study on cytopenic-phenotype MF (6). Furthermore, patients with *ASXL1* mutations frequently exhibit rapidly progressing PMF (31, 32), and accelerated-phase and blast-phase MPNs (33) are commonly associated with *ASXL1* mutation (34). Therefore, ASXL1 inhibitors are potential therapeutic agents for inhibiting cytopenic MF (35). In patients experiencing progressive thrombocytopenia, bone marrow biopsy could be considered to check for leukemic transformation or plan hematopoietic stem cell transplantation at a young age for better survival outcomes.

Our study has some limitations. First, the relatively small number of patients might affect the generalizability of the findings. However, the genomic profile of the entire cohort was available, and the platelet count time series was evaluated with a relatively long-term follow-up. Second, this was a single-center study that could have introduced selection bias. Thus, further prospective multi-center studies with larger cohorts are required to validate the results. However, treatment protocols were similar for all patients, even during the long-term follow-up; therefore, we could investigate genomic and immunologic traits. Comparative prospective studies of thrombocytopenia groups and the effect on survival outcomes may provide more comprehensive insights.

In conclusion, our study demonstrated that dynamic thrombocytopenia is a predictive factor for the survival of patients with MF. Furthermore, thrombocytopenia dynamics are crucial among patients with MF with normal platelet counts at diagnosis. We also identified *ASXL1* mutations and CD45RA^+^CD4^+^ T cells as possible predictors for cytopenic MF. These results indicate that leukemic transformation can be prevented and survival outcomes of patients with MF can be improved by targeting the clones with a cytopenic phenotype.

## Data Availability

The data that support the findings of this study are available in the Dryad at http://datadryad.org/stash/share/aWHHTNrKZxwHy6UNgp2cf6edHF7XXNvJiKm_BGZXr6U. DOI: 10.5061/dryad.v15dv425r. The authors confirm that the data supporting the findings of this study are available within the article and its Supplementary materials.
