# Supplementary material for: Genetic and immunologic features associated with thrombocytopenia progression and poor prognosis in patients with myelofibrosis
Source: Front Med (Lausanne). 2024 Nov 7;11:1461421. doi: 10.3389/fmed.2024.1461421 (PMC11580038; doi:10.3389/fmed.2024.1461421)
Supplement: Supplementary file 1 [file Data_Sheet_1.PDF]

Supplementary Material

Supplementary Table S1. Flow cytometry analysis of lymphocytes according to thrombocytopenia subgroups

	PLT≥100 (n = 41)	PROG (n = 32)	PLT<100 (n = 12)	PLT≥100 vs. PROG, *p*	PROG vs. PLT<100, *p*	PLT≥100 vs. PLT<100, *p*
Frequency (%), mean ± SE						
$CD3^{+}$	23.1 ± 13.0	22.8 ± 15.0	27.9 ± 19.5	0.938	0.363	0.321
$CD3^{+}CD4^{+}$	9.5 ± 7.0	7.9 ± 6.0	7.7 ± 5.6	0.294	0.939	0.419
$CD3^{+}CD8^{+}$	10.0 ± 6.2	9.1± 8.2	13.5 ± 18.3	0.625	0.438	0.52
Regulatory T	8.6 ± 7.1	6.9 ± 5.4	7.4 ± 5.3	0.301	0.781	0.614
HLA $DR^{+}CD4^{+}$	2.6 ± 2.7	3.5 ± 4.2	4.4 ± 4.5	0.322	0.511	0.21
HLA $DR^{+}CD8^{+}$	0.9 ± 1.0	1.2 ± 1.6	1.2 ± 1.5	0.372	0.969	0.4
$CD4^{+bright}$	11.0 ± 8.1	8.7 ± 6.6	7.6 ± 6.2	0.187	0.619	0.178
$CD4^{+dim}$	4.5 ± 3.8	6.3 ± 5.6	8.1 ± 6.6	0.134	0.364	0.094
$CD45RA^{+}CD4^{+}$	17.8 ± 11.2	11.8 ± 8.5	11.5 ± 10.7	0.014	0.919	0.089
$CD45RA^{+}CD8^{+}$	17.8 ± 11.0	16.9 ± 10.5	16.8 ± 10.2	0.74	0.966	0.78
$CD3^{+}CD4^{bright}$	3.1 ± 5.1	7.0 ± 8.7	6.3 ± 9.5	0.029	0.815	0.284
Ratio of $CD4^{dim}$-to-$CD4^{bright}$*	0.9 ± 2.1	1.4 ± 1.8	2.4 ± 4.3	0.062	0.576	0.027

PLT≥100, platelet count of ≥100 × 10^{9}/L; PROG, progression to a platelet count less than 100 × 10^{9}/L; PLT<100, platelet count of <100 × 10^{9}/L; SE, standard error

* Comparison of the $CD4^{dim}$-to-$CD4^{bright}$ ratio using the Mann–Whitney *U* test.

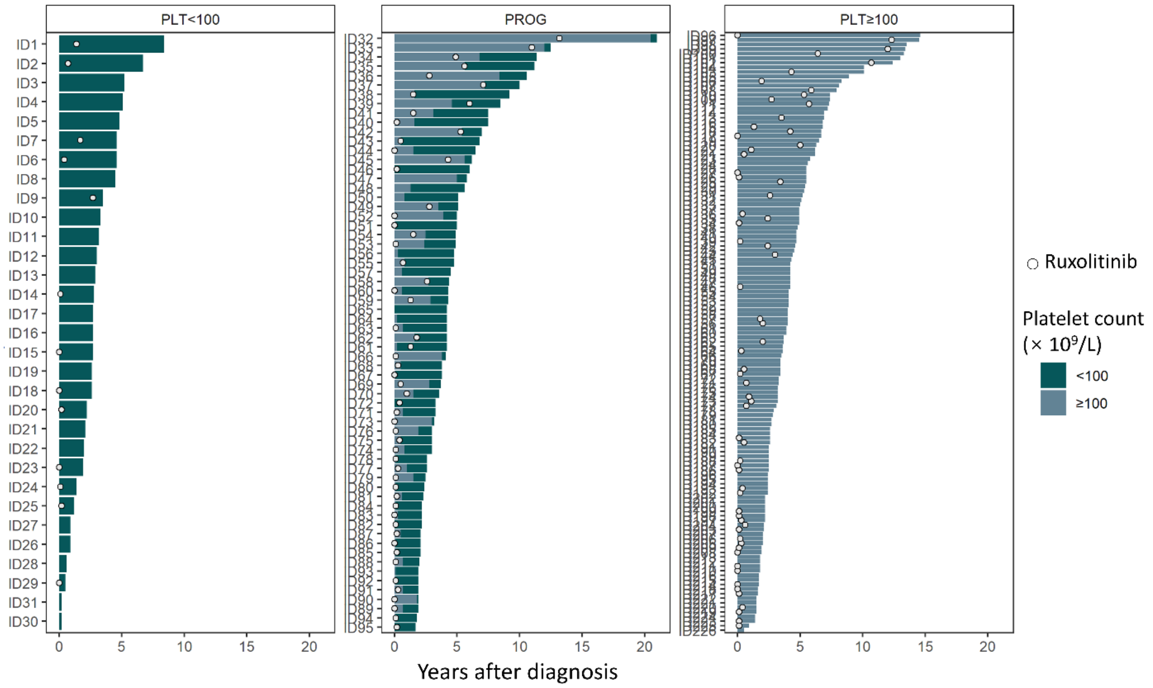

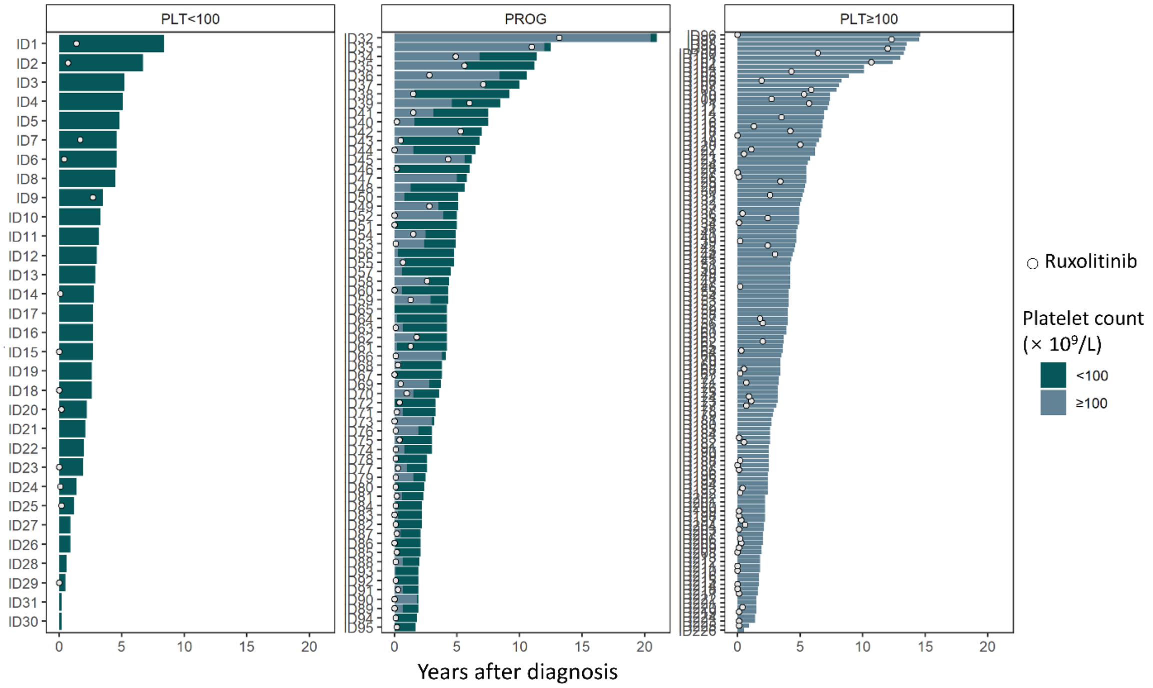

Supplementary Figure S1. Swimmer's plot describing the time points of changes in platelet count threshold by platelet subgroups.

PLT≥100 group, with a platelet count of 100 × 10^9/L or higher at diagnosis; PROG group, indicating progression to a platelet count of <100 × 10^9/L during follow-up; PLT<100 group, with a platelet count of < 100 × 10^9/L at diagnosis.

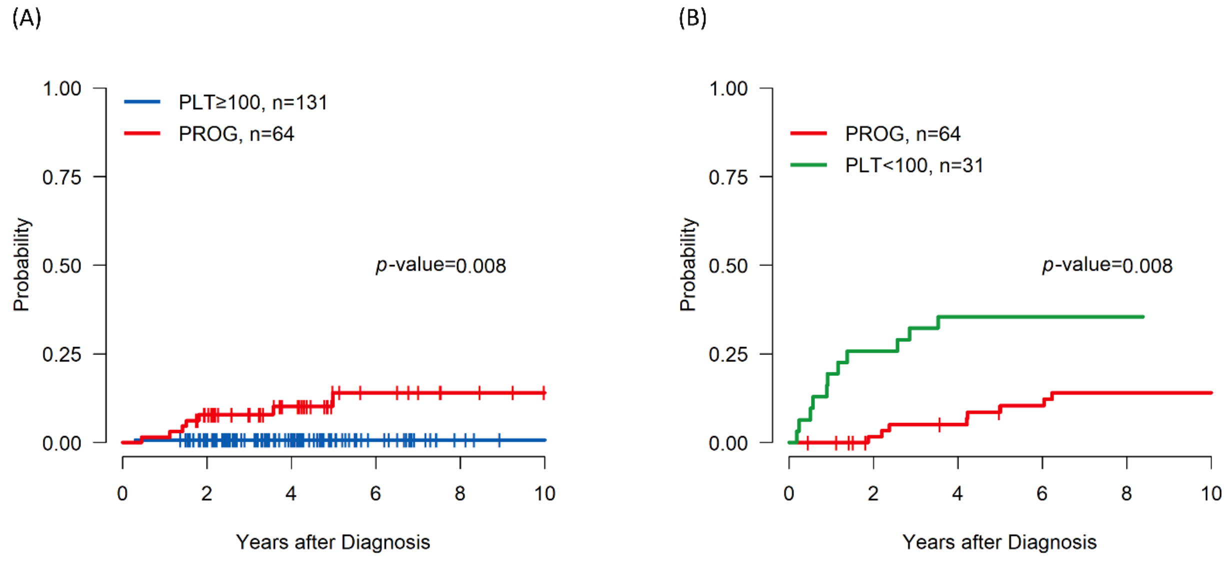

Supplementary Figure S2. Cumulative incidence of leukemic transformation and non-leukemic mortality in patients with myelofibrosis according to platelet subgroup.

(A) Cumulative incidence of leukemic transformation in the platelet count ≥ 100 × 10^9/L (PLT≥100) and progression to a platelet count of <100 × 10^9/L (PROG); (B) non-leukemic mortality in patients with myelofibrosis in the platelet count < 100 × 10^9/L (PLT<100) and PROG groups.

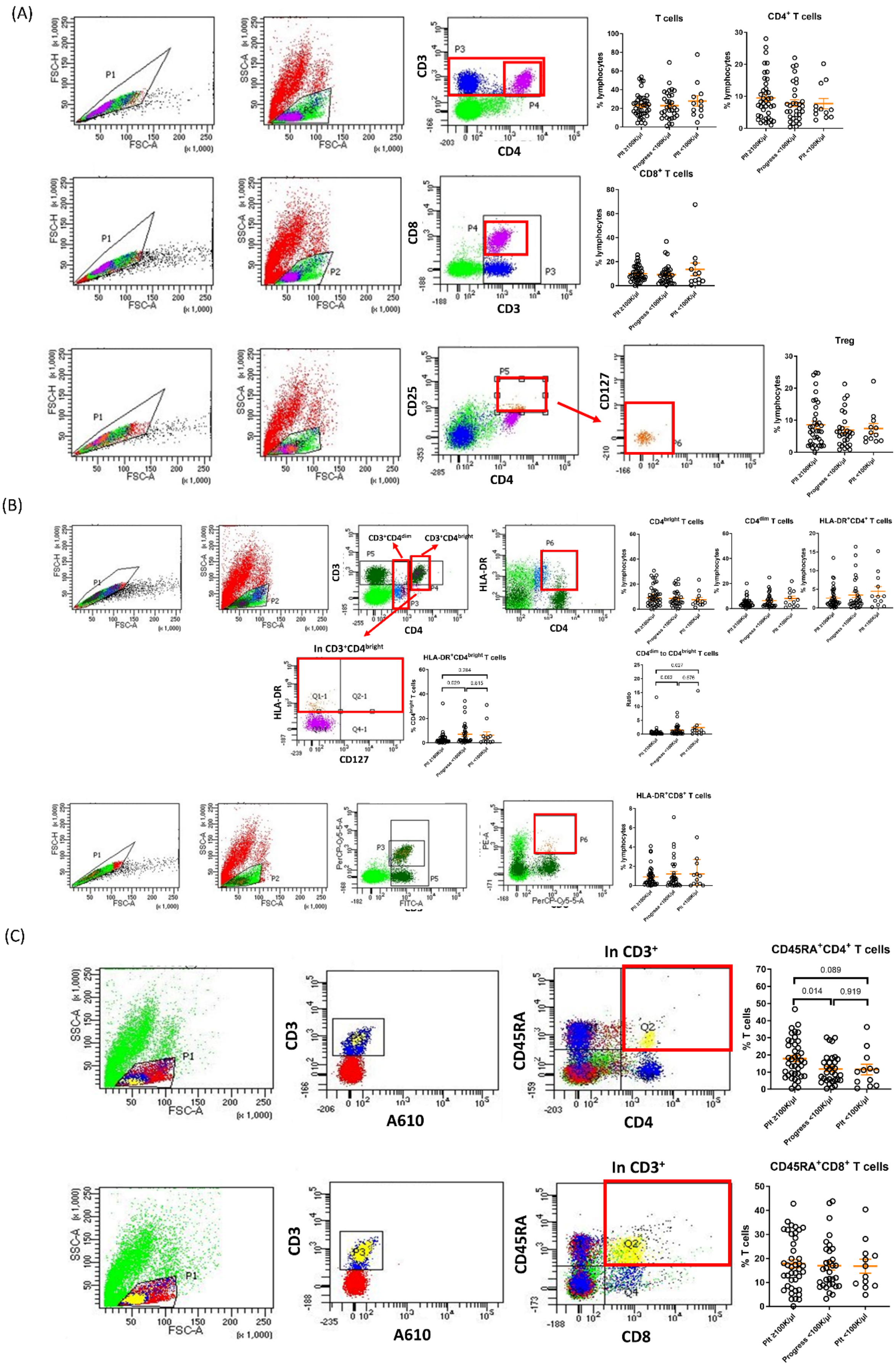
(A)
FSC-H
FSC-A
SSC-A
P1
P2
P3
P4
CD3
CD4
T cells
CD4+ T cells
% lymphocytes
Plt ≥100K/µl
Progress <100K/µl
Plt <100K/µl
CD8
CD8+ T cells
CD25
P5
CD127
P6
Treg
(B)
CD3+CD4dim
CD3+CD4bright
HLA-DR
CD4bright T cells
CD4dim T cells
HLA-DR+CD4+ T cells
In CD3+CD4bright
Q1-1
Q2-1
Q4-1
HLA-DR+CD4bright T cells
% CD4bright T cells
0.284
0.020
0.615
CD4dim to CD4bright T cells
Ratio
0.027
0.002
0.676
HLA-DR+CD8+ T cells
PerCP-Cy5-5-A
FITC-A
PE-A
(C)
A610
In CD3+
CD45RA
Q2
Q4
CD45RA+CD4+ T cells
% T cells
0.089
0.014
0.919
CD45RA+CD8+ T cells

Supplementary Figure S3. Gating strategy and percentage of T-cell subtypes according to thrombocytopenia subgroups.

(A) Total T cells, CD4+, CD8+, and Treg cells; (B) CD4bright, CD4dim, HLA–DR+CD4+, HLA–DR+CD4bright, the ratio of CD4dim-to-CD4bright, and HLA–DR+CD8+ T cells; (C) CD45RA+CD4+ and CD45RA+CD8+ T cells. Orange horizontal lines and error bars indicate the mean and standard error, respectively.
